# Precision medicine in Parkinson’s disease: emerging treatments for genetic Parkinson’s disease

**DOI:** 10.1007/s00415-020-09705-7

**Published:** 2020-01-23

**Authors:** Susanne A. Schneider, Roy N. Alcalay

**Affiliations:** 1 Department of Neurology, Ludwig-Maximilians-University of München, Marchioninistr. 15, 81377 Munich, Germany; 2 Department of Neurology, Columbia University Medical Center, New York, NY USA

**Keywords:** Genetic Parkinson’s disease, SNCA, GBA, LRRK2, Kinase inhibitor, Small molecule compounds, Venglustat, Ambroxol, Clinical trial, TORC1 inhibitor, Superprecision medicine

## Abstract

In recent years, numerous clinical trials for disease modification in Parkinson’s disease (PD) have failed, possibly because of a “one-size-fits all” approach. Alternatively, a precision medicine approach, which customises treatments based on patients’ individual genotype, may help reach disease modification. Here, we review clinical trials that target genetic forms of PD, i.e., *GBA*-associated and *LRRK2*-associated PD. In summary, six ongoing studies which explicitely recruit *GBA*-PD patients, and two studies which recruit *LRRK2*-PD patients, were identified. Available data on mechanisms of action, study design, and challenges of therapeutic trials are discussed.

Parkinson’s disease (PD) is the second most common neurodegenerative disorder, affecting more than 6 million people worldwide [1]. Numerous drugs for the treatment of PD are avilable on the market. While drugs targeting the dopaminergic pathway treat motor symptoms, there is no evidence that they modify disease progression. This “one-size-fits all” approach may very well explain why clinical trials for disease modification in PD have failed. Treatments that target the underlying pathophysiology are required. Since the pathophysiology of PD may be different in different patients, studies should be designed that assess PD treatment on a more individual basis. Therefore, a precision medicine approach in PD is very timely.

## Precision medicine—a conceptual framework

Precision medicine—also referred to as personalized medicine or individualized medicine—aims to tailor the specific treatment for the right person at the right time. To achieve this, it uses diagnostic tools to identify specific biomarkers, often genetic, to help assess which medical treatments will be best for each patient [2]. By combining data from these tests with a patient’s medical history and important factors influencing health status, targeted prevention and treatment plans can hopefully be developed in the future [2]. Thus, precision medicine is not a new concept. It is a conceptual framework which became a hot topic beyond the medical sphere when President Obama announced a research initiative that aims to accelerate progress toward a new era of precision medicine in 2015 [3]. There are various benefits of precision medicine, including the detection of disease onset at the earliest moment and thereby shifting the emphasis in medicine from reaction to prevention (Table 1).

**Table 1:** Benefits of precision medicine [2]

Diagnose disease more accurately
Select optimal therapies and target medicines and dosages precisely
Increase safety, reduce adverse drug reactions
Detect onset of disease at the earliest moment, incl. detection of early subclinical correlates of neuronal death prior to clinical (motor) manifestation
Shift emphasis in medicine from reaction to prevention (i.e., protection of neurons to avoid neuroal death)
Increase the efficiency of the health system by improving quality

In the context of neurodegenerative disorders, at the time of clinical manifestation (and certainly at the time of diagnosis), a substantial number of neuros has been permanently lost. Hence, early detection of at-risk individuals will be instrumental for early treatments (which will ideally protect the cells from neuronal death). Sucessful precision medicine will thus move from current reactive approaches to early detection, protection and prevention. Early detection of individuals at-risk to develop neurodegenerative disorders is a major challenge. However, in the case of genetic subforms, early detection is feasible by confirming their genetic status with a minimal-invasive test.

## The genetic architecture of Parkinson’s disease

In the past decade, we have seen incredible progress in elucidating the genetic architecture of PD: over a dozen Mendelian loci are known to cause familial PD. In addition, multiple loci have been identified by genome-wide association studies (GWAS) that are mostly associated with a small increase in risk of PD. These latter genetic variations of weak effect strength may occur as commonly as 40% in the general population, but convey only a mildly (up to ~ 1.5-fold) increased disease risk [4]. Even when combining all risk factors, the odds ratio is only 3–4 (i.e. there is a 3 to 4 times increased risk of developing the disease) [5]. Notably, alteration in the same gene may lead to different variants and mutations with differrent risk association with PD [6]. For example, some point mutations in *LRRK2* are causative for PD, while coding polymorphisms in the gene are strong risk factors and additional higher frequency variants at the *LRRK2* locus contribute to a small increase in risk of developing PD [7].

A recent meta-analysis suggested that the detectible heritable component of PD (based on genome-wide SNPs and less significant SNPs included in a polygenic risk score) is around 20% [8]. There is compelling evidence of yet-to-be-discovered additional genetic factors that contribute to the etiology of PD. In addition, environmental risk factors are yet to be discovered.

This tremendous progress in understanding the genetic architecture has set the ground for the development of treatments based on disease mechanism rather than symptoms. Here, we will review clinical trials which target genetic forms of PD, i.e., explicitly recruit (or enrich for) patients with a genetic form of PD.

## Parkinsonism associated with *GBA* mutations

Homozygous mutations in the glucocerebrosidase (*GBA*) gene cause Gaucher disease (GD), the most common autosomal recessive lysosomal storage disease, with an estimated annual incidence of 1/60,000 and an estimated carrier frequency [9] of 0.7–0.8% in the general population. Some ethnicities show higher mutation rates; specifically, in the Ashkenazi Jewish (AJ) population, there is an annual incidence is 1/1,000 and carrier frequencies as high as 6% in all AJ.

The clinical presentation of GD can be divided into systemic, which are present in all forms of GD, and include hepatosplenomegaly, painful skeletal disorders and pancytopenia, and neurological manifestations, which are present in the more severe types of GD, GD-II and GD-III. Both GD patients and healthy heterozygous carriers are at increased risk for PD [10] and longterm follow-up showed worsening in motor and non-motor prodromal PD features [11]. *GBA* mutations are a common risk for PD and are present in 7–10% of PD patients worldwide. Among Ashkenazi Jews, around 20% of PD patients carry a *GBA* mutation [12]. High prevalences have also been reported in the Netherlands, where 15% of PD patients carry a *GBA* mutation (oral communication, Dana Hilt). The lowest carrier frequency was reported to be 2.3% in Norwegian Parkinson's disease patients [13]. Notably, there is considerable reduction of penetrance in that only about 10% of GBA carriers will develop PD (which is however considerably higher compared to the global PD prevalence of 1–2% of the general population aged 65 years or older) and studies suggest that penetrance is age-dependent [14].

Clinically, *GBA* heterozygotes may be indistinguishable from iPD. However, they may have an earlier age at onset, more prevalent cognitive impairment and may not respond to levodopa as well as iPD patients [15, 16]. *GBA* mutations are also associated with other alpha-synucleinopathies, including DLB [17] (pathologically confirmed) and in some, but not all studies, with MSA [18-22]. In contrast, there was no association with essential tremor or AD (Alzheimer’s disease).

More than 300 mutations in *GBA* have been reported [23], some with milder (e.g., the N370S mutation), others with more severe (e.g., the L444P mutation) biological consequences and clinical presentations (e.g., age at onset or progression rate). The encoded protein, glucocerebrosidase, is a lysosomal enzyme which plays a role in the breakdown of glucocerebroside into glucose and ceramide. In GD, there is lysosomal build-up of the substrate glucocerebroside in the reticulo-endothelial system with reduced clearance capacities.

Pathologically, the brains of patients with heterozygous *GBA* mutations strongly resemble those from patients with iPD. However, there is also widespread cortical Lewy body involvement in *GBA* mutation carriers [16, 22]. A few studies showed a reciprocal relationship between levels of glucocerebrosidase (Gcase; the enzyme encoded by *GBA*) and levels of the aggregate-prone protein alpha-synuclein [24]. Notably, iPD patients also have reduced GCase activity (about 33% decrease) in the substantia nigra and cerebellum, making treatments that target *GBA* relevant for iPD and patients with PD dementia as well [21].

While the PD field can benefit from decades of research done for GD, the underlying mechanisms of how exactly *GBA* leads to the development of PD are not fully understood. One of the hypotheses is that there is a self-propagating bidirectional feedback loop between GCase and a-synuclein. On the one hand, loss of GCase activity causes a-synuclein accumulation and oligomerization, resulting in neurotoxicity through aggregate-dependent mechanisms [25]. Furthermore, elevated a-synuclein inhibits lysosomal maturation and normal GCase activity. a-synuclein hinders GCase transport from the endoplasmic reticulum to the lysosome. This continues over time until the threshold for neurodegeneration is reached [25].

**Fig. 1:**
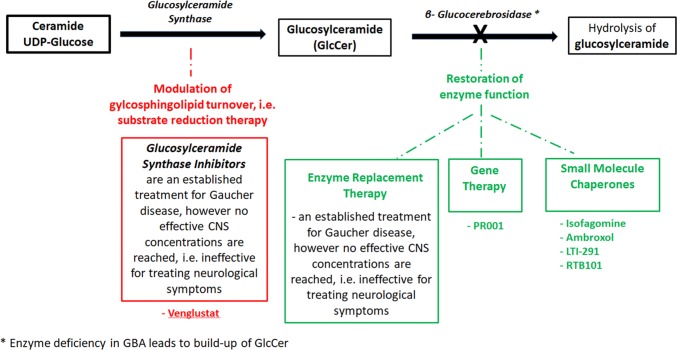
Treatment approaches for GBA-associated PD include modulation of gylcosphingolipid turnover and restoration of enzyme function

Based on this, targeted treatments can take different approaches including modulation of gylcosphingolipid turnover and restoration of enzyme function (Table 2; Fig. 1).

**Table 2:** GBA-targeting treatments for PD in the clinical phase aiming at modulation of glycosphingolipid turnover and restoration of enzyme function

GBA	MOVES-PD study Part 1	MOVES-PD study Part 2	AiM-PD			
Compound	Venglustat (GZ/SAR402671)		Ambroxol	RTB101	LTI-291	PR001
Administration	Oral		Oral	Oral	Oral	Injections
Sponsor	Sanofi		UCL and Cure PD Trust	Restorbio	LTI/Allergan	Prevail
RCT No	NCT02906020		NCT02941822		(NL7061; NTR7299)^a^	
Mechanism	Glucosylceramide synthase inhibiton; reduction of GBA-related GSLs		GCase activation	TORC1 inhibition	GCase activation	Gene therapy, AAV-based
Status	Completed	Recruiting, estimated primary completion 2021	Estimated completion 04-2018	Ongoing; data expected 2020	Recruiting in Leiden (NL)	Clinical centers initiated
Phase	2		2a	1b/2a	1b	1b
Design	Multicenter, randomized, double-blind, placebo–controlled, sequential cohort	Prospective, single-centre, open label	Multicenter 2:1 randomized double-blind, placebo-controlled	Multicenter, 2:1 randomized, double-blind, placebo-controlled	Randomized, placebo-controlled, double-blind, parallel study	Randomized, double-blind, sham procedure-controlled, ascending dose study
Total *N* of part	17		10 + 10	45	Apprx. 40	30/16
GBA-PD	$\surd$		$\surd$	$\surd$	$\surd$	$\surd$
Idiopathic PD	No		$\surd$	$\surd$	No	$\surd$
Age	18–80 yrs (mean 58 yrs)		40–80 yrs		18 years or older	
Duration	36 weeks	52 weeks + 104 weeks extension	6 months	4 weeks	28 days	
Doses tested	3 escalating doses	1 dose	5 escalating doses	300 mg; ± sirolimus	10 or 60 mg once daily	Two escalating dose cohorts

^*a*^see https://www.trialregister.nl/trial/7061 for more information

### Treatment directed at modulation of gylcosphingolipid turnover

Substrate reduction therapy inihibits the biosynthesis of lipid subtrates and thereby prevents their accumulation. While this approach does not target the mutant gene or dysfunctional enzyme itself, it is an effective FDA approved treatment of the systemic symptoms of Gaucher disease. However, the approved inhibitors show no effective CNS concentration and do not affect the neurological symptoms of Gaucher disease (i.e., Gaucher type II and III). However, new glucosylceramide synthase inhibitors, show good brain penetration and improved a-synuclein processing and behavioural outcomes in mouse models [26, 27]. Based on these findings, Sanofi launched the MOVES-PD study, a randomized, double-blind, placebo-controlled trial, to assess the efficacy and safety of the glycosylsynthase inhibitor Venglustat (GZ/SAR402671). Initial results from a phase I study were recently published [28]. Briefly, 17 *GBA*-PD were enrolled (13 on venglustat, 4 on placebo; mean age 58 years, mean disease duration 7 years) into this 36-week randomized placebo-controlled double-blind sequential cohort study of once daily venglustat at three escalating doses. No serious adverse events occurred. Side effects included psychological, neurological and gastrointestinal-related symptoms. Plasma and cerebrospinal fluid (CSF) glucosylceramide levels decreased in a dose-dependent manner (up to 75%). This favorable safety and tolerability profile of venglustat at all doses led the company to advance to a phase II, a 52-week trial which is currently ongoing [28].

### Treatment directed at restoration of enzyme function

Other therapies focus on restoration of enzyme function, thus increasing glucocerebrosidase activity, especially in the brain. This can be achieved by (1) enzyme-replacement therapy (ERT) with recombinant glucocerebrosidase. This treatment is available for patients with Gaucher disease. However, as in the case of the currently approved substrate reduction therapies, ERT does not cross the blood–brain barrier and does cannot affect the neurological symptoms found in GD Type II and III. Of note, there are no data on the use of ERT in PD.

Another approach would be (2) gene therapy using adeno-associated virus vectors to deliver engineered DNA to human cells [27, 29]. As for *GBA*, preclinical studies in mice demonstrated that adeno-associated virus-mediated expression of glucocerebrosidase corrected the aberrant accumulation of the toxic lipid glucosylsphingosine and reduced the levels of ubiquitin, tau, and alpha-synuclein aggregates [30]. Prevail Therapeutics, a new company launched in 2017, aims to start clinical trials with PR001 in 16 GBA-PD patients in late 2019 [31]. The company will also test the compound in children with neuropathic Gaucher disease starting in the first half of 2020.

Furthermore, (3) small molecules have attracted attention [32]. Early glucocerebrosidase chaperones that underwent clinical trials for Gaucher disease included isofagomine (afegostat‐tartrate, AT2101). This treatment did not lead to significant clinical improvement, and further clinical development for this indication was discontinued [27].

Ambroxol, which is a promising small molecule chaperone widely used in Europe as a mucolytic agent, may potentially facilitate the transit of the misfolded GCase protein to the lysosome [33]. Ambroxol has been shown to improve lysosomal function and increase enzyme activity in *in-vitro* studies utilizing dermal fibroblasts with *GBA1* mutations [34] as well as in studies performed on non-human primates (i.e., cynomolgus monkeys) with *GBA1* mutations [35]. The effects of ambroxol at high doses are currently being studied in the AiM-PD study, sponsored by UCL and the Cure Parkinson’s Trust, UK [36]. Twenty PD patients (10 GBA-positive & 10 GBA-negative status) are treated with up to 420 mg/day (which is considerably higher compared to 30–120 mg used for the treatment of respiratory disease) in order to evaluate the safety, tolerability and pharmacodynamics of ambroxol at five escalating doses. Outcome measures include clinical assessments of motor and cognitive function as well as blood and CSF biomarkers. As mentioned above, GCase activity is also reduced in iPD patients’ brains (SN) [37], making the therapy potentially relevant for iPD. The effect of ambroxol in non-GBA-PD [36] and non-GBA-PD dementia [38] will be better understood once results from the two ongoing studies become available which include ten non-GBA-PD and 75 PDD patients [38, 39] (treated at a daily doses of 420 mg / day or 525–1050 mg/day, respectively).

The effects of the activator of the GCase enzyme LTI-291 were studied in a one-month phase 1b trial conducted in the Netherlands, where the rate of *GBA* mutations are reported to be around 15%. Around 40 *GBA*-PD patients participated. There were no safety events and data showed a good dose-dependent brain penetration (personal communication). The company, Lysosomal Therapeutics, Inc. (LTI), is a Massachusetts-based biotech venture, which plans to develop therapies for Gaucher disease and other lysosome-based neurodegenerative diseases.

Small molecules have also targeted PD by modifying *GBA*-independent pathways. One such example is RTB101, which is an inhibitor of target of rapamycin complex 1 (TORC1) [40]. Rapamycin, which is known for its immunosuppressant properties, prolongs lifespan by 15–25% in various non-mammalian organisms, even when given late in life; it has also been found to increase health-span. A five year-study in dogs is planned to test geroprotective effects of RTB101 in mammals [41]. Rapamycin reached public attention when Bloomberg magazine publicized it as the potential “forever pill” on its cover in 2015, which reflects the great desire of rejuvenation. TORC1 plays a role in cell growth, aging and is the switch between fasting and feeding states [40]. Mutations in TORC1 cause focal cortical dysplasia, an established cause of epilepsy. The role of mTORC1 in regulating autophagy may also have implications for neurodegenerative diseases. In preclinical models, TORC1 inhibition induces autophagy and prevents neuronal loss [41, 42]. It improves motor function in multiple PD models including a-synuclein transgenic mice and MPTP models of PD [43]. In oncology cell cultures, treatment with RTB101 reduced the levels of glucosylceramide, the main substrate of GCase. A current phase 1b/2a trial of RTB101 in combination with sirolimus involved 45 PD patients with or without *GBA* mutation. The study was initiated in early 2019; data are expected in 2020.

## *LRRK2*-associated Parkinsonism: kinase inhibitors are a promosing target

**Fig. 2:**
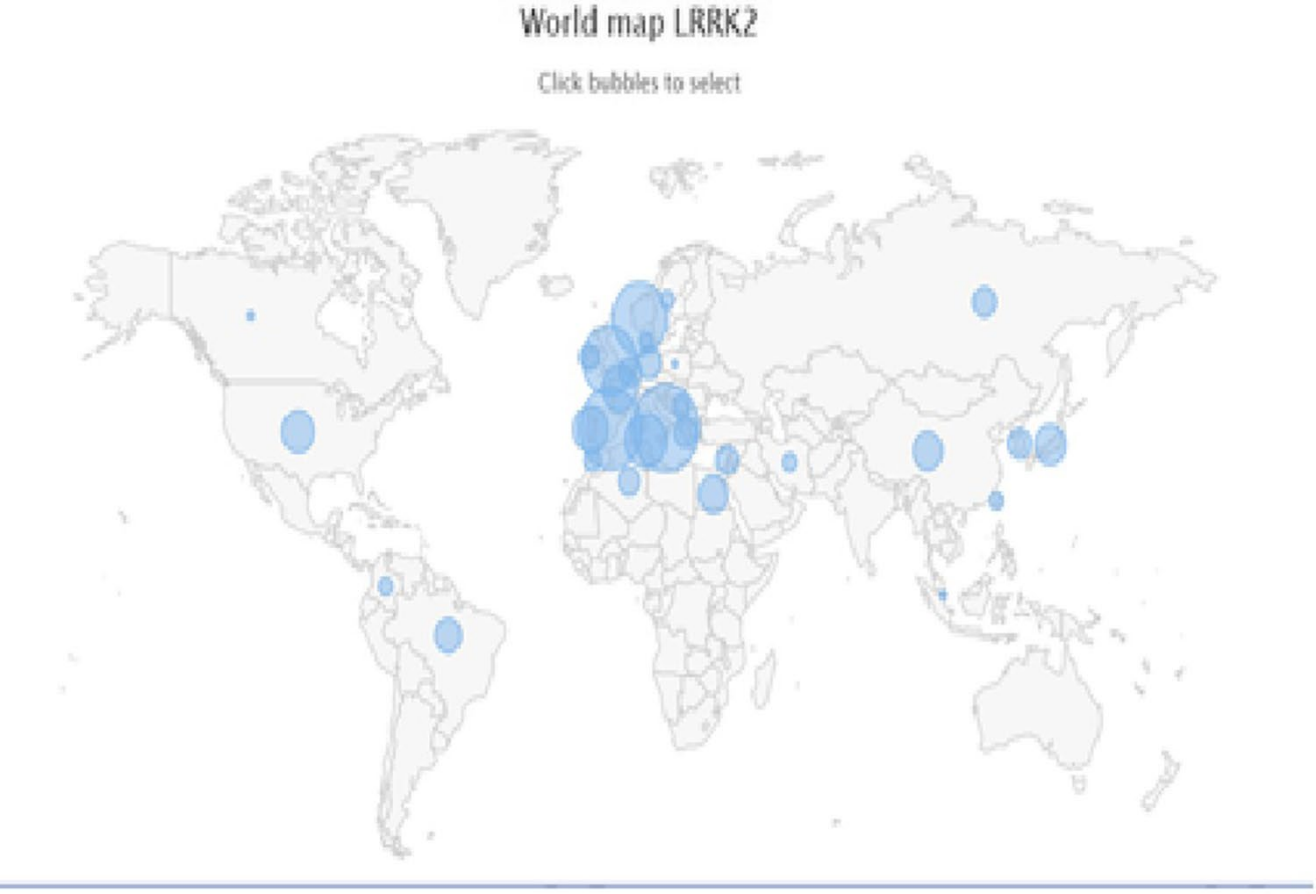
World map of LRRK2-associated Parkinsonism. 533 cases have been reported. Circles reflect frequency per region. Data and image were retrieved from the MDSGene Website [47]

*LRRK2* mutations are the most common cause of autosomal dominant PD accounting for 5–15% of dominant familial PD and 1–3% of sporadic PD (Fig. 2). The International *LRRK2* Consortium study estimated that the most common mutation in *LRRK2*, G2019S, alone accounts for 1% of sporadic and 4% of familial PD patients [44].

Similar to *GBA*, mutations in *LRRK2* are more common in certain ethnicities. North African Arabs (mutation-positive: 36% in familial, 39% in sporadic PD) and Ashkenazi Jews (28% in familial, 10% in sporadic PD) have the highest frequencies. As in *GBA*, different mutations and variants confer different levels of risk for PD. For example, the G2019S mutation, which is common in Whites, confers a higher risk for PD than the common Asian variants, G2385R and R1628P [11, 13]. These latter two variants are detected in around 5–10% of Asian PD patients [45]. The G2385R variant is associated with an odds ratio of 2.24. Penetrance in *LRRK2* is age-dependent and estimations in the general population are widely variable, ranging between 30 and 74% [46, 47].

Additionally, non-coding polymorphisms close to the *LRRK2* locus act as risk factors for sporadic disease [48] Furthermore, *LRRK2* interacts with the protein products of at least two GWAS hits, RAB7L1 and GAK, linking PD-related genes with monogenic and complex forms [49, 50].

*LRRK2* is a large gene with 51 exons, spanning a genomic region of 144 kb. It contains five functional domains including a leucine rich repeat domain. More than 80 missense mutations have been reported, but only around one dozen are pathogenic [48]. The mechanisms by which mutations cause PD have not been completely disentangled yet, but there is increasing evidence of increased LRRK2 kinase function in PD. The G2019S mutation, for example, results in a direct two-to-threefold increase in kinase activity [51, 52]. Others studies have focused on the GTP domain which may also play an important role. Loss of function, on the other hand, i.e., by haploinsufficiency of LRRK1 or LRRK2, appears to be neither a cause of nor protective against PD [53].

The potential gain-of-function effect would be an attractive target for treatment because inhibition is easier to achieve than improvement of reduced protein activity (as in *GBA*). Furthermore, kinase inhibitors are widely used in oncology, and the PD field can benefit from such achievements in other fields. Since the first generation of LRRK2 inhibitors, newer compounds have progressively improved in potency, selectivity, and brain penetrance. However, efficacy and safety remain a concern. That is because other tissues, particularly the kidney, lung, and a subtype of peripheral immune cells, robustly express LRRK2. For example, the kidney has a ~ 6.2-fold higher expression of LRRK2 compared to the brain [52]. This is a potential source of peripheral side effects, which can include abnormal accumulations, a-syn aggregations, and impaired autophagy-lysosomal function induced by LRRK2 inhibitors [53-55]. More recent data, however, suggest that compounds that only partially downregulate LRRK2 levels or kinase activity, i.e., by 50% or less, are unlikely to produce major side effects related to on-target safety [56] and lipid droplets in lamelar bodies are absored after the drug is withdrawn. One alternative to avoid systemic toxicity is to find a way to specifically modify LRRK2 activity in the brain without modifying activity peripherally. Several strucutrally different LRRK2 inhibitors from Genentech, GSK, Merck and Pfizer are in the pipeline (Table 3) [27]. The compound developed by Denali is already in clinical trial. A phase 1b trial in healthy individuals has been completed, which included pulmonary and renal safety parameters. The company is advancing DNL201 (GNE-7915) to a Phase 1b safety and biomarker study in LRRK2-linked PD and iPD. 30 mild to moderately affected PD patients with or without *LRRK2* mutation will be randomized to low or high dose DNL201 or placebo in this 28-day randomized placebo-controlled trial. The first patient was reported in December 2018; data readout is expected for the end of 2019. To facilitate recruitment, a “direct-to-consumer” approach for testing and counselling will be available [57]–a strategy that proved successful in genetic testing with the PPMI initiative. Most recently, Denali has announced a strategic collaboration with a gene diagnostic lab, Centogene, in order to globally identify and recruit *LRRK2* mutation carriers, further characterize this genetic subtype, and build a source for patient recruitment for future studies [58]. However, such strategies of a commercial diagnostic lab in concert with a drug company to offer directed to consumer diagnostic testing is viewed critically by some.

**Table 3:** LRRK2-targeted treatments including LRRK2 inihibitors and antisense oligomeres under development for PD

LRRK2	Denali trial				
Compound	DNL-201	No public data	No public data	No public data	BIIB094
Sponsor	Denali	GSK	Pfizer	Genetech	Biogen
RCT No	NCT03710707				NCT03976349
Mechanism	LRRK2 inhibition	LRRK2 inhibition	LRRK2 inhibition	LRRK2 inhibition	Antisense oligomere
Status	Ongoing, recruiting, data expected end of 2019	Planned	Under development	Under development	Ongoing
Phase	1b	N/a	N/a	N/a	Phase 1
Design	Multicenter, randomized, placebo-controlled	N/a	N/a	N/a	
Total *N* of pat	30	N/a	N/a	N/a	62
LRRK2-PD	$\surd$	N/a	N/a	N/a	$\surd$
Idiopathic PD	$\surd$	N/a	N/a	N/a	$\surd$
Age	30–75	N/a	N/a	N/a	35–80
Duration	28 days	N/a	N/a	N/a	N.d.
Doses tested	Low / High	N/a	N/a	N/a	Single-and multiple-ascending-dose

Finally, Biogen is currently recruiting LRRK2 patients into one arm of a phase 1 trial. These patients will receive a single intrathecal injection of the compound BIIB094, an antisense oligomere (ASO), on multiple days. Recently, ASOs have produced a lot of interst in a variety of disorders, including spinal muscular atrophy, Huntington’s disease or non-neurological disorders such as cancers [59, 60]. ASOs reduce the expression of a mutated gene by binding to target mRNAs and blocking the translation of the abnormal protein or inducing its degradation [60]. ASOs can also promote splicing around mutations. For disorders due to toxic gain-of-function such as LRRK2, further investigation regarding ASOs is warranted. In a preclinical study, administration of LRRK2 ASOs to the brains of mice reduced LRRK2 protein levels and fibril-induced α-syn inclusions [61]; data from humans are not yet available.

Interestingly, most recent studies found an mechanistic and therapeutic convergence of LRRK2 and GCase with reduced GCase activity in dopaminergic neurons derived from PD patients with LRRK2 mutations and increased GCase activity induced by inhibition of LRRK2 kinase activity [62]. Rab10 was identified as a key mediator of LRRK2 regulation of GCase activity and may be an interesting target for future studies [62].

## Final remarks

Recent failures in large Phase III clinical trials for PD suggest that disease modification would be difficult to achieve as long as we treat PD as one disease one pathophysiology. Therefore, we believe that precision medicine in PD may be a promising alternative.

As summarized in this review, several gene-targeted therapies are being tested in clinical trials and numerous more are in the pipeline. These are exciting times. However, the process of bringing a drug into the clinic is cumbersome [63]. Pharmaceutical Research and Manufacturers of America (PhRMA) estaimate that for every 5000–10,000 compounds screened, only 250 enter preclinical testing, five enter human clinical trials, and one is approved by the Food and Drug Administration, with only two in ten drugs generating enough revenue to recoup their research and development costs [64]. Thus, set-backs will not come unexpected.

In addition to the hurdles of all clinical trials, precision medicine trials may be more complicated. It is unclear who may benefit from precision medicine drugs. Would these be useful only for mutation carriers (and therefore require an orphan drug assignment) or would they be beneficial for the larger group of idiopathic PD or atypical parkinsonian disorders? It seems unlikely that all these patients will respond to the same drug. Indeed, even within the group of *GBA* mutation carriers, one may have to differentiate due to the effect that a specific mutation has on the protein. For example, the affinity of chaperones to a mutated enzyme may be different depending on the mutation. Furthermore, a new drug that facilitates protein function may fail in patients with null mutations who do not express any protein. The term “superprecision medicine” has been used to capture this phenomenon.

There are challenges that remain that need to be overcome when planning or conducting a clinical trial (Table 4) [65]. A major challenge will be to recruit a large enough number of study participants. Genotyping significantly larger proportions of PD patients would be required. Different strategies have been developed for this purpose, including the Parkinson’s Foundation effort called PD GENE and the “direct-to-consumer” approach, which may allow identification of eligible individuals even if they do not live close to a movement disorders unit. Raising awareness and educating the community, including physicians, patients and caregivers, will be an important step to reach critical numbers. We are hopeful that the treatment for PD will drastically change in the next decade and evolve beyond dopaminergic or surgical treatments.

**Table 4:** Top four challenges and barriers to effective clinical trials as perceived by health professionals, patients, and their caregivers—for full list see [65]

Scientists and other health professionals	Patients and caregivers
Lack of funding	Risk of potential adverse consequences and potential side effects
Lack of administrative support and time available to manage the trial	Disruption to normal medication regimen
Slow and difficult recruitment of people	Prospect of receiving a placebo instead of the active drug
Lack of practical support	Upheaval and inconvenience to life that trial participation would cause

Another challenge is the relative lack of biomarkers that reliably reflect disease progression and response to treatment. This applies to genetic subtypes as well as the larger group of iPD in general. Concerted efforts are being made to identify a biospecimen-based (i.e., blood, urine, CSF or biopsy), imaging, or other (e.g., electrophyiological) biomarker of PD or disease progression. Among these, the PPMI initiative is a valuable source that brings together longitudinal data and specimen collection from more than 1200 volunteers with PD [66].

Advancing precision medicine will further encourage and support the next generation of scientists to develop creative new approaches for detecting, measuring, and analyzing a wide range of biomedical information—including molecular, genomic, cellular, clinical, behavioral, physiological, and environmental parameters [3].
